# Butoxyethanol: A Novel and Exceptionally Strong Eluent for ICPMS Detection Eliminating the Organic Mode

**DOI:** 10.1021/acs.analchem.6c04370

**Published:** 2026-09-10

**Authors:** Julia Truschner, Walter Goessler, Bassam Lajin

**Affiliations:** † Institute of Chemistry, Analytical Chemistry for the Health and Environment, 27267 University of Graz, Universitaetsplatz 1, 8010 Graz, Austria; ‡ Institute of Chemistry, ChromICP, University of Graz, Universitaetsplatz 1, 8010 Graz, Austria

## Abstract

Inductively coupled plasma mass spectrometry (ICPMS) is gaining increasing recognition for speciation analysis of nonmetals, but many compounds, such as those containing halogens and sulfur, are highly hydrophobic, requiring the use of the organic ICPMS mode to address compatibility with reversed-phase liquid chromatography. Herein, we introduce 2-butoxyethanol (BE) as a novel eluent with exceptional elution strength and plasma tolerability. BE allows operation under standard conditions used with 100% aqueous mobile phases, with no plasma shutdown or carbon buildup. Direct comparison with methanol when used under the organic ICPMS mode showed superior sensitivity by 3-5-fold despite the employment of highly optimized and mild organic mode conditions. BE concentrations ≤30 v/v remarkably provided an elution strength matching that of 90–100% v/v methanol, which was demonstrated in multiple proof-of-concept applications involving the elution of highly hydrophobic analytes from a reversed-phase C18 column. These included separation of a complex mixture of polychlorinated biphenyls (PCBs) where superior resolution and unique chromatographic selectivity were found, as well as arsenolipid screening in seaweed, where highly complex profiles in various edible species were observed, revealing >100 signals at instrumental LOD down to 6 ng As L^–1^ (20 μL injection volume). The introduced eluent presents a significant advancement over previous work, including yet stronger elution than that achievable with 1,2 hexanediol at lower column backpressure, providing a major step forward in optimizing the practice of reversed-phase liquid chromatography to suit the changing demands of speciation analysis.

## Graphical abstract

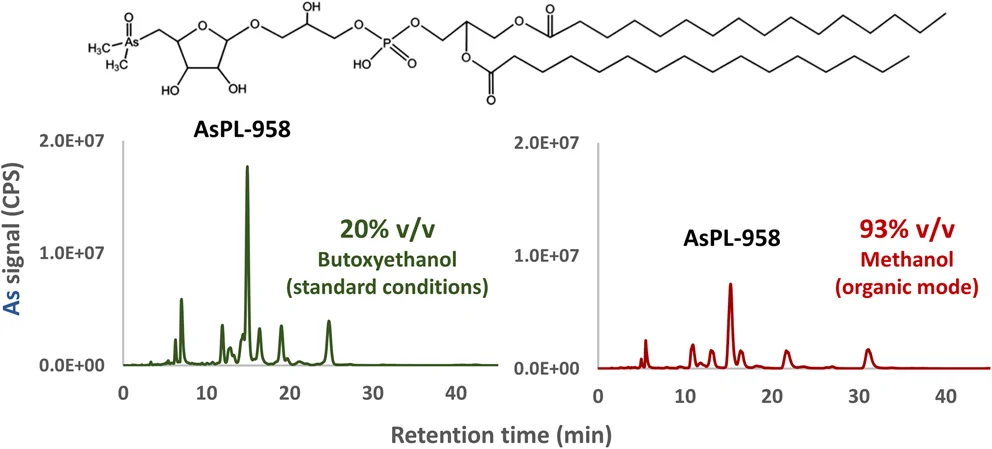

## 1 Introduction

The advent of tandem mass spectrometry to inductively coupled plasma mass spectrometry (ICPMS/MS) greatly expanded the range of elements amenable to speciation analysis to include key elements of biological and environmental interest such as sulfur, chlorine, and phosphorus.^1,2^ Chromatographic separation with ICPMS detection (HPLC-ICPMS) provides a complementary dimension to molecular mass spectrometry in targeted and nontargeted analysis.^3,4^ Particularly promising novel areas of application may include environmental screening for emerging chlorinated persistent organic pollutants (POPs), identification of novel sulfur-containing natural products, and phospholipid profiling. Analytes involved in such applications are however highly hydrophobic, which would necessitate the use of the so-called organic ICPMS mode to enable compatibility with reversed-phase liquid chromatography, as carbon is poorly tolerated by the inductively coupled plasma, resulting in shutdown and sensitivity loss at high concentrations. Therefore, to fully exploit the potential of the technique in various areas of research, there have been increasing demands for tailoring the general practice of liquid chromatography to the special needs of ICPMS detection in parallel with the changing importance of the technique associated with the expansion of its capabilities to previously refractory elements.^1^

Although the tolerability of organic solvents by the plasma is a well-recognized and thoroughly studied topic,^5^ research within the theme of introducing novel eluents that fall within an area of intersection between desirable properties for both ICPMS detection and chromatographic separation has been scarce. We have been actively working on introducing novel chromatographic approaches to address the compatibility issue between ICPMS and reversed-phase liquid chromatography.^6,7^ Notably, we introduced hexanediol as a novel eluent, allowing strong elution and high compatibility.^8,9^ However, the high viscosity of hexanediol due to the diol functional group hinders its application with long UHPLC columns, which would be necessary to tackle the diversity of the elemental metabolomes in complex matrices. Furthermore, there is an ongoing demand for eluents with different functional groups and chemical structures to expand the arsenal of eluents available to address challenging chromatographic separations. Moreover, there is still a need for even stronger eluents to enable fast elution and high-throughput analysis for exceptionally hydrophobic analytes such as highly halogenated POPs and high-molecular-weight lipids.

In this work, we introduce butoxyethanol as a novel eluent previously not employed in liquid chromatography, and we directly compare this eluent with hexanediol as well as methanol under the ICPMS organic mode to demonstrate its advantages, cementing the significance of these emerging eluents in speciation analysis.

## 2 Experimental Section

### 2.1 Chromatographic Conditions

All chromatographic experiments were performed with a YMC Triart C18 UHPLC column, 150 mm × 2.1 mm, 1.9 μm (YMC Europe GmbH, Germany), with a mobile phase flow rate of 0.3 mL min^–1^, column temperature of 60 °C, and injection volume of 20 μL. The organic solvents used for mobile phase preparation consisted of variable proportions of methanol (LC-MS grade, purity ≥99%), 1,2-hexanediol (purity 98%), and 2-butoxyethanol (purity ≥99%), all including a final concentration of 0.1% v/v formic acid (ACS grade, purity >98%). All solvents and reagents were obtained from Sigma-Aldrich, Steinheim, Germany, except pure water, which was produced in-house using a Milli-Q water purification system (18.2 MΩ cm, Merck Millipore GmbH, Vienna, Austria). Chromatographic separation was performed on an Agilent 1290 Infinity II UHPLC system (Agilent, Waldbronn, Germany).

### 2.2 ICPMS/MS Setup and Parameters

An Agilent 8900 ICPQQQ system was coupled with the chromatographic system using an AriMist polyether ether ketone (PEEK) nebulizer. Butoxyethanol and hexanediol were used under standard conditions, where the ICPMS/MS system was equipped with a glass double-pass spray chamber, a nickel/copper sampler and skimmer cones, and a quartz plasma torch with an injector inner diameter of 2.5 mm. Key parameters included RF power 1550 W, RF matching 1.6 V, total carrier gas flow 0.85–1.0 L min^–1^ (including nebulizer gas flow of 0.65 L min^–1^ in addition to argon makeup gas), plasma gas 15.0 L min^–1^, auxiliary gas 1.0 L min^–1^, sampling depth 6.0 mm, and spray chamber 2°C.

Due to poor plasma tolerability, experiments involving methanol were performed under the organic ICPMS mode while applying the above instrumental parameters with modifications, namely, the replacement of nickel/copper sampler cone with a platinum cone, the use of a plasma torch with narrow inner diameter (1.5 mm), RF power 1600 W, RF matching 1.8 V, spray chamber temperature −5 °C, and adding oxygen as optional gas to the plasma at 5–15% depending on methanol concentration. The total carrier gas flow was optimized for each methanol concentration within the range of 0.6–0.8 L min^–1^. To obtain the maximum possible sensitivity under the organic ICPMS mode, flow splitting and post-column dilution were completely avoided, which was enabled by using a microflow chromatographic column (2.1 mm ID) at 0.3 mL min^–1^ flow rate.

All studied elements were detected using 0.2 mL min^–1^ oxygen as reaction gas and monitored at *m*/*z* M^+^ → M^+^ + 16, except for chlorine, where hydrogen reaction gas was used at 3.0 mL min^–1^ and detection was performed at *m*/*z* 35→37.

### 2.3 Sensitivity Testing

A systematic investigation of the impact of butoxyethanol on sensitivity and how it compares with other solvents was performed under both ICPMS and molecular mass spectrometry, with the latter being relevant for applications involving simultaneous coupling between the two detectors for structural identification. For testing sensitivity under ICPMS, inorganic standard solutions of six elements (P, As, S, Se, Cl, and Br) were monitored under flow injection by using the chromatographic conditions described in previous sections.

For molecular mass spectrometry, investigation was based on a range of compounds with variable ionization properties in the positive- and negative-ion mode where applicable (arsenobetaine, arsenocholine, glyphosate, ethephone, dimethylarsinic acid, esmolol, phosphoserine, diclofenac, and ascorbic acid). This was performed on an Agilent triple quadrupole Ultivo ESIMS/MS system using the following general source settings: nebulizer gas temperature and flow 350 °C and 10 L min^–1^; sheath gas temperature and flow 400 °C and 12 L min^–1^; nebulizer pressure 35 psi; capillary voltage 3000 V. Multiple replicates (3–6) were used to ensure repeatability within RSD 10%.

### 2.4 Proof-of-Concept Applications

The elution behavior under butoxyethanol was investigated and compared with methanol and hexanediol for the separation of the highly hydrophobic polychlorinated biphenyl in a commercially available mixture of 19 congeners (PCB-1, -5, -18, -31, -44, -52, -66, -87, -101, -110, -138, -141, -151, -153, -170, -180, -183, -187, and -206) (10 mg L^–1^ (molecular concentration), LGC, Wesel, Germany). The chlorine count in these congeners was within the range of 1–9, with log *K*_ow_ values within 4.5–8.2.^10^

A further application involved arsenolipid profiling in six commercially available edible seaweed species (*Himanthalia elongate*, *Undaria pinnatifida*, *Neopyropia yezoensis*, *Saccharina japonica*, *Saccharina latissima*, and *Gracilariopsis lemaneiformis*). Lipid extracts were prepared based on the Matyash method.^11^ Briefly, 0.5 g of freeze-dried and homogenized seaweed sample was extracted with a mixture of 1.5 mL of methanol and 4.5 mL of methyl *tert*-butyl ether (MTBE) using a cross-shaker and sonication at room temperature. Water (1.5 mL) was added to minimize hydrophilic arsenic species, and then the organic phase was dried in a rotary evaporator, and the residue was reconstituted in 1.0 mL of methanol and transferred to amber HPLC glass vials.

To demonstrate the utility of butoxyethanol in nontargeted analysis applications, arsenolipids in seaweed samples were identified using identical chromatographic conditions under ICPMS/MS and high-resolution mass spectrometric detection based on an electrospray ionization-quadrupole time-of-flight (ESI-qTOF) mass analyzer (Agilent 6546 qTOF) operated at experimental parameters similar to those in the previous section. Fragmentation data and exact mass measurements (mass accuracy ±1–5 ppm) were used for molecular formula assignment.

## 3 Results and Discussion

Butoxyethanol displayed exceptional elution strength, as low concentrations of ≤30% v/v matched 90-100% methanol for elution of arsenolipids and polychlorinated biphenyls used as examples of highly hydrophobic analytes (Figure 1). Furthermore, the novel eluent significantly surpassed hexanediol (Figure 1), a strong eluent that was previously tested against and found superior to all organic chromatographic eluents in common use, such as methanol, acetonitrile, and isopropanol.^9^ In particular, the separation of arsenolipids displayed striking effects. As little as 20% v/v butoxyethanol was superior to the elution strength of 93% methanol and 35% hexanediol (Figure 1D–F). These results indicate that butoxyethanol is applicable for exceptionally hydrophobic compounds and, when used at higher concentrations, would be unmatched by eluents in common use, enabling elution and detection of hydrophobic compounds that may escape detection under common eluents.

**Figure 1:**
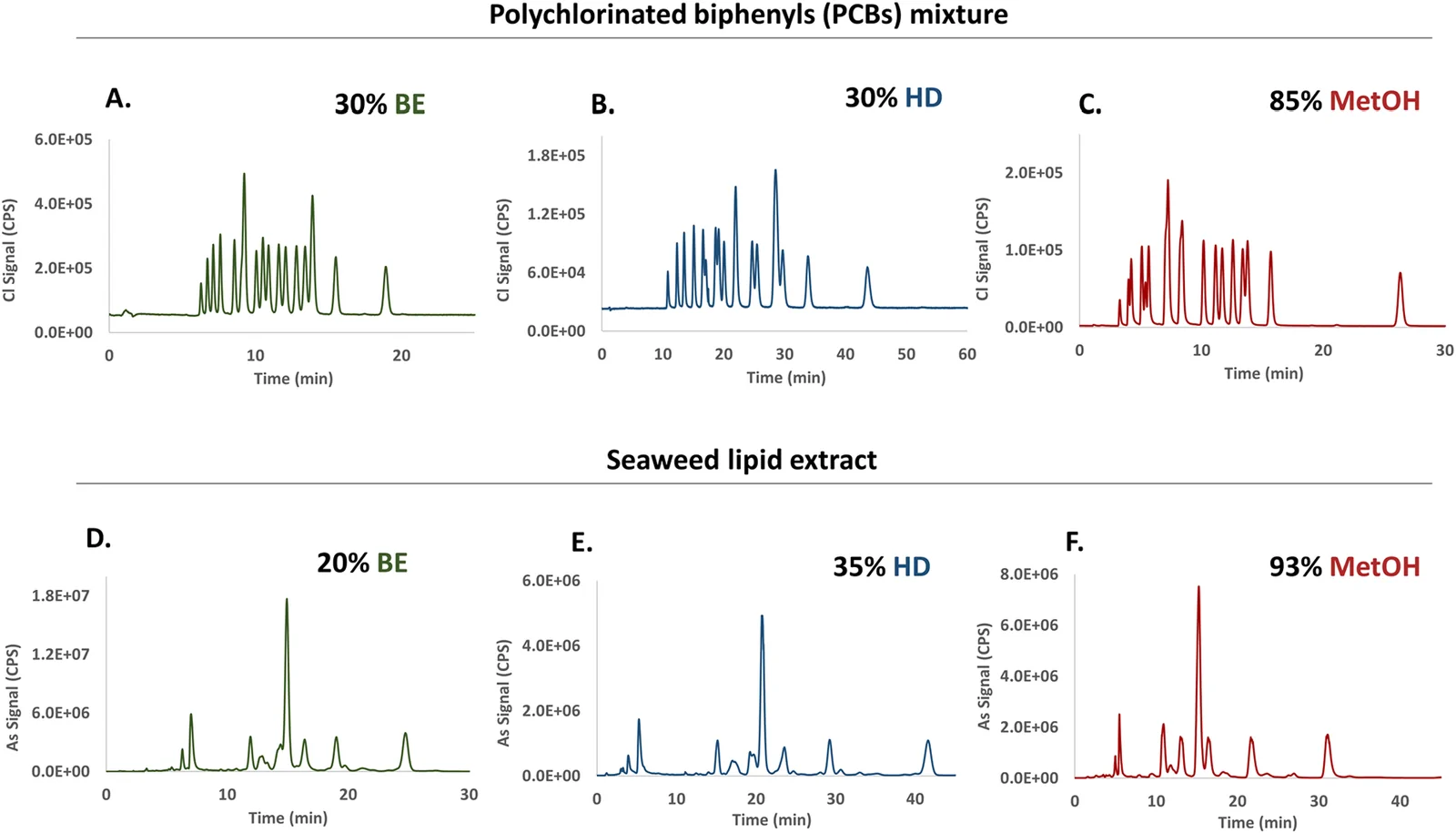
Comparing chromatographic separation between butoxyethanol (BE), hexanediol (HD), and methanol (MetOH) for separation of a mixture of polychlorinated biphenyls (PCB) with chlorine count 1–9 (A–C) and arsenolipids in Wakame seaweed extract (D–F). Note the stronger elution at lower concentrations with BE compared with the other solvents, particularly methanol, as well as the different selectivity under BE and superior separation within a shorter time frame for the PCB mixture (chlorine count 1–9, see the Section 2).

Besides stronger elution, butoxyethanol has the advantage over hexanediol of significantly lower viscosity (3.3 mPa s for butoxyethanol vs. ca. 87 mPa s for 1,2-hexanediol at 20 °C), which enabled the use of maximum available length of a UHPLC C18 column (150 mm × 2.1 mm, 1.9 μm) at backpressure ca. 800 bars (30% v/v concentration, 0.3 mL min^–1^ flow rate, and 60°C column temperature). The combination of stronger elution and lower viscosity means that matching between butoxyethanol and hexanediol would require operating at >2-fold higher column backpressures under hexanediol, which renders the use of UHPLC columns problematic.

The observed strong elution behavior is a result of multiple facets unique to this solvent over common eluents. First, butoxyethanol has an amphiphilic structure and displays micelle formation with a critical micelle formation at ca. 12% v/v,^12^ which can enhance elution through strong solvation. Second, the amphiphilic and linear structure of butoxyethanol results in strong adsorption on the linear C18 carbon chain, which results in coating the stationary phase with highly polar hydroxyl groups, which greatly decreases its overall hydrophobicity. This mechanism is similarly displayed by hexanediol but is more pronounced due to the higher hydrophobicity of butoxyethanol (log *P* 0.83 ^13^) than that of 1,2-hexanediol (log *P* 0.58 ^14^). Indeed, this mechanism appears to result in a significant influence on chromatographic selectivity and resolution, as it is interesting to note superior separation within a short time frame in combination with a “gradient-like” peak spacing under butoxyethanol relative to methanol (Figure 1A–C). Therefore, butoxyethanol can serve as an additional general tool that can be attempted for tackling separation challenges in liquid chromatography in general.

Although the presence of carbon can enhance the signal of some elements such as arsenic, selenium, and phosphorus due to the carbon enhancement effect,^15^ excessive carbon concentrations result in plasma cooling and lower ionization efficiency for most elements, especially those with high ionization potential such as the halogens. To achieve maximum sensitivity and exploit the full potential of ICPMS/MS, it is desirable to utilize low concentrations of organic solvents in reversed-phase liquid chromatography when coupled with ICPMS detection, not only to avoid plasma cooling/shutdown and carbon buildup but also to remain as close as possible to the optimal range for maximum carbon enhancement effect for relevant elements (5–15% v/v) and minimize carbon load for those elements with high ionization potential.

Furthermore, current practice involved in the organic ICPMS mode can decrease sensitivity, particularly when very poorly tolerated solvents (e.g., acetonitrile) are used with high flow rates that necessitate more aggressive settings. When compared with methanol at similar concentrations within the range of 5.0–30% v/v, butoxyethanol showed up to 2-fold higher peak sensitivity (Figure 2). While this concentration range is appropriate for the elution of most hydrophobic compounds with butoxyethanol, matching elution strength using methanol would require applying significantly higher concentrations, as discussed above. Because of plasma instability, sensitivity under these high concentrations of methanol was studied in the organic ICPMS mode. Despite using highly optimized conditions including the use of a narrow-bore chromatographic column with low flow rate and completely avoiding flow splitting and post-column dilution, the overall gap in sensitivity between 5.0–30% butoxyethanol and 60–100% methanol, which would be equivalent in terms of elution strength, was within 3–5-fold (Figure 2). The use of higher flow rates and/or acetonitrile would necessitate more aggressive conditions of the organic mode, which can widen this gap in sensitivity to >10-fold.

**Figure 2:**
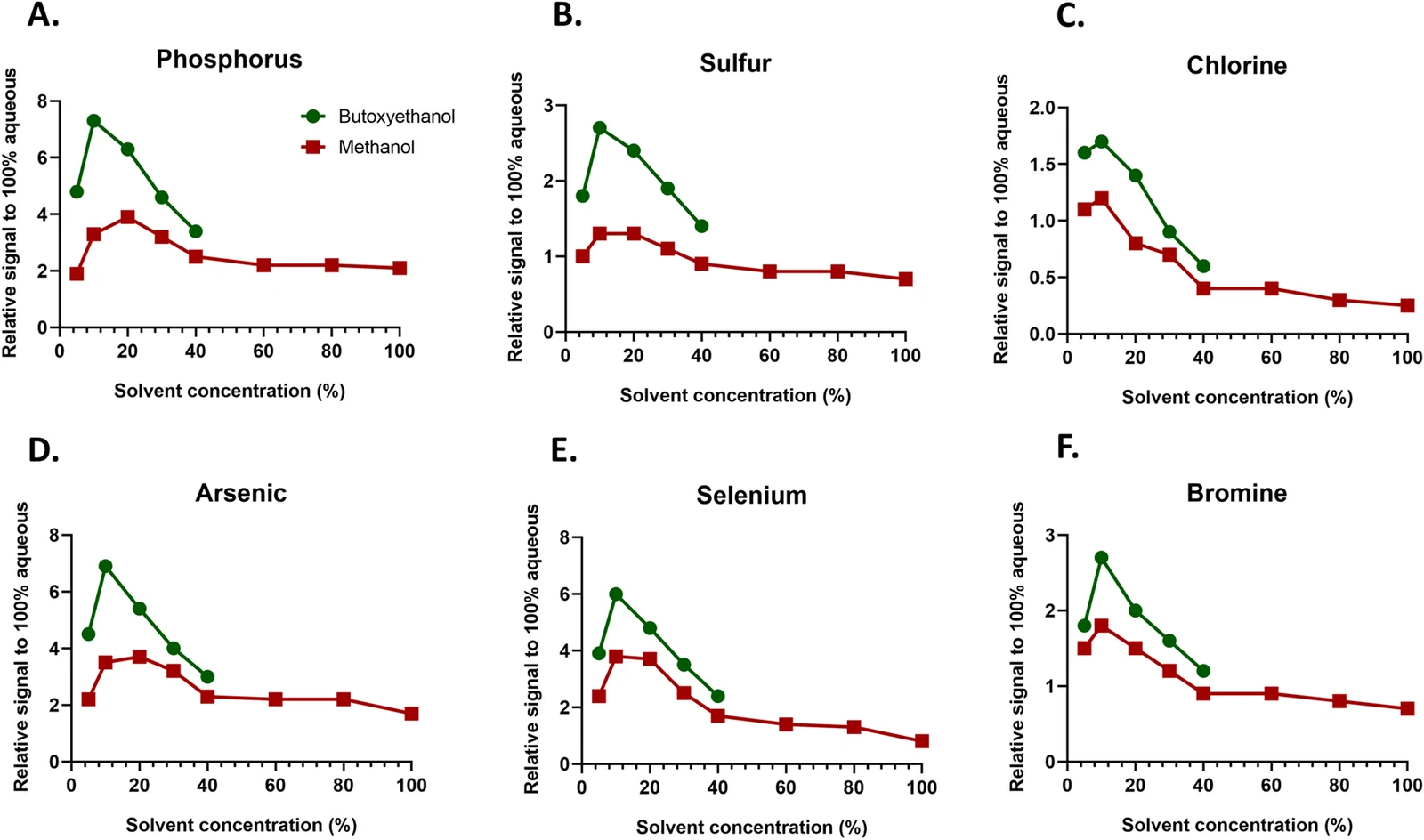
Impact of butoxyethanol (BE) on ICPMS sensitivity and how it compares with methanol (MetOH), for different elements (phosphorus (A), sulfur (B), chlorine (C), arsenic (D), selenium (E), and bromine (F)). Note that due to poor tolerability with MetOH and to extend comparisons to chromatographically equivalent concentrations, the organic ICPMS mode was used for MetOH experiments, whereas experiments under butoxyethanol were performed under standard conditions (see the Section 2). Sensitivity was normalized to 100% aqueous serving as a reference point, and conditions were optimized for each mobile phase composition. Note that chromatographically relevant comparisons must be based on comparing 10–30% v/v BE with 70–100% MetOH. This shows a 3–5-fold gap in sensitivity despite avoiding aggressive organic mode conditions (see text), which would be necessary at higher mobile phase flow rates and/or using acetonitrile as an eluent.

Typical instrumental limits of detection for arsenolipids have been reported in the range of 1–10 μg As L^–1^,^16,17^ which is >10-fold higher than those achievable for hydrophilic arsenic species that are detected under standard conditions.^18,19^ As a proof of concept, we tested the applicability of butoxyethanol for studying the diversity in the arsenolipid profiles in six common edible seaweed species commercially available (Figure 3). The achieved instrumental limit of detection was 6–30 ng As L^–1^ (depending on peak width, injection volume 20 μL), which is on par with the lowest limits of detection achievable for arsenic speciation with ICPMS/MS detection^20^ and >20-fold lower than previous studies involving arsenolipid profiling using the organic ICPMS mode.^16,17^ This resulted in the appearance of highly complex arsenolipid profiles, with >100 peaks detected in the investigated samples in total (Figure 3).

**Figure 3:**
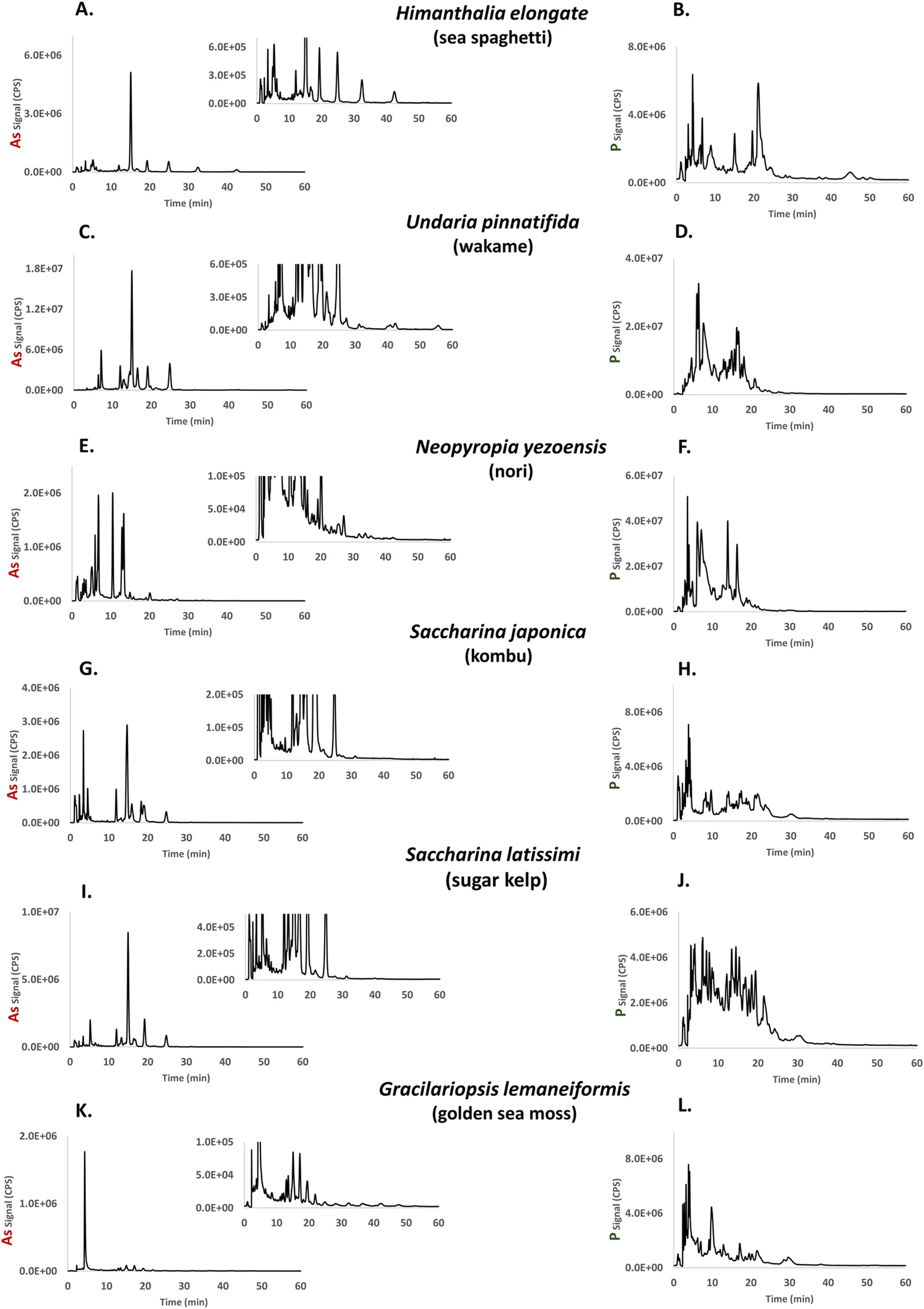
Simultaneous arseno/phospholipid profiling with HPLC-ICPMS/MS in six edible commercially available seaweed samples (directly indicated on graph (A, C, E, G, I, K for arsenic and B, D, F, H, J, L for phosphorus)). The mobile phase consisted of 20% v/v butoxyethanol with 0.1% formic acid (see the Section 2). Molecular mass spectrometry revealed that the most common form of arsenolipids in seaweed (i.e., arsenosugar phospholipids (As-PL)) elutes within the region of 10–30 min under this chromatographic system (Figure 4). Arsenic hydrocarbons/fatty acids as well as monoacyl As-PL elute within the region of 1–10 min. The insets show magnified chromatograms revealing extremely complex profiles that may escape detection under less sensitive experimental set-ups. The LOD was 6–30 ng As L^–1^ (depending on peak width) in the present work, which is lower than the typical instrumental LOD for arsenolipids, 1–1.0 μg As L^–1^.

Butoxyethanol has not been previously used as an eluent for liquid chromatography and is therefore not commercially available as an HPLC- or LCMS-grade solvent. When compared with LCMS-grade methanol, the employed commercially available butoxyethanol showed comparable background signals for the six studied elements, which are relevant for speciation analysis, with chlorine as a notable exception with 10-fold higher background. Furthermore, we demonstrated the utility of butoxyethanol with ESIMS detection by employing this eluent for coupling with an electrospray ionization molecular tandem mass spectrometric detector (ESIMS/MS), and it was possible to identify arsenosugar phospholipids detected under this chromatographic system (Figure 3I) in the positive-ion mode at ESIMS/MS limits of detection down to 25 ng As L^–1^ (injection volume 5.0 μL). Major members of this class in sugar kelp (*Saccharina latissimi*), taken as an example, included AsPL-958 (RT 15 min, 0.48 mg As kg ^–1^, dry mass basis), AsPl-986 (RT 19 min, 0.14 mg As kg ^–1^), AsL-930 (RT 12 min, 0.06 mg As kg ^–1^), and AsL-1014 (RT 25 min, 0.09 mg As kg ^–1^) (Figure 4), which is in line with previous reports.^21^ Further systematic testing to assess the effects of butoxyethanol on ESIMS sensitivity was performed using a variety of compounds. While sensitivity under ESIMS detection was found comparable with methanol in the negative-ion mode, 2–5-fold lower sensitivity was observed in the positive-ion mode relative to equal concentrations of methanol for some of the compounds tested at high butoxyethanol concentrations (Figure 5).

**Figure 4:**
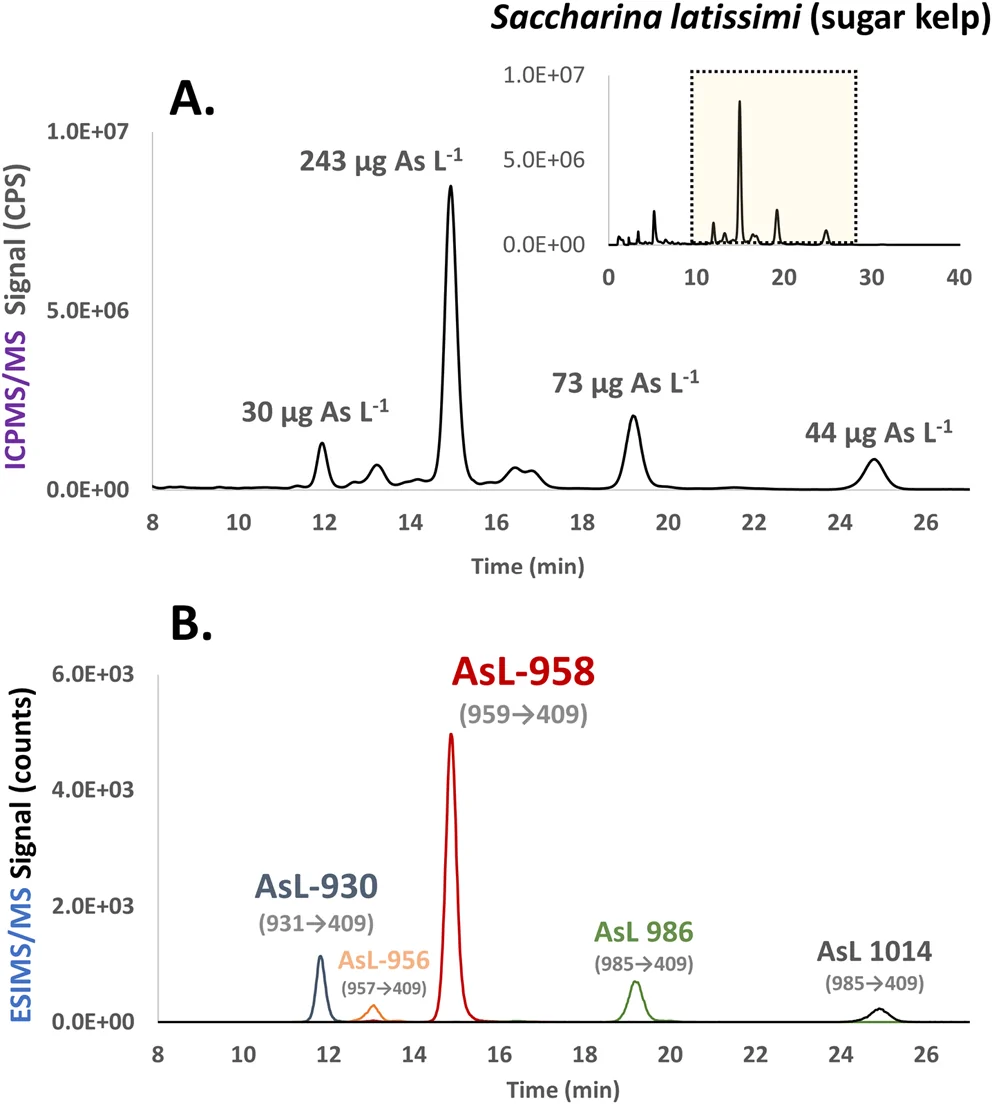
Identification of arsenosugar phospholipids in sugar kelp (*Saccharina latissima*) with LC-ESIMS/MS. To demonstrate the applicability of butoxyethanol for identification by molecular mass spectrometry, common arsenosugar phospholipids were detected with an Ultivo Triple quadrupole mass spectrometer (B) under identical chromatographic conditions used with ICPMS/MS detection (A). Quantitative data in the lipid extract was based on ICPMS/MS, and based on that, the S/N ratio was calculated under ESIMS detection with LOD (S/N 3) estimated at ca. 25 ng As L^–1^ (injection volume 5 μL). Identification was based on precursor ion scan using the characteristic product ion at *m*/*z* 409 corresponding to the dimethylarsinoyl ribose headgroup and guided by the ICPMS/MS trace. The mobile phase consisted of 20% BE v/v with 0.1% formic acid. For further chromatographic and mass spectrometric conditions, see the Section 2.

**Figure 5:**
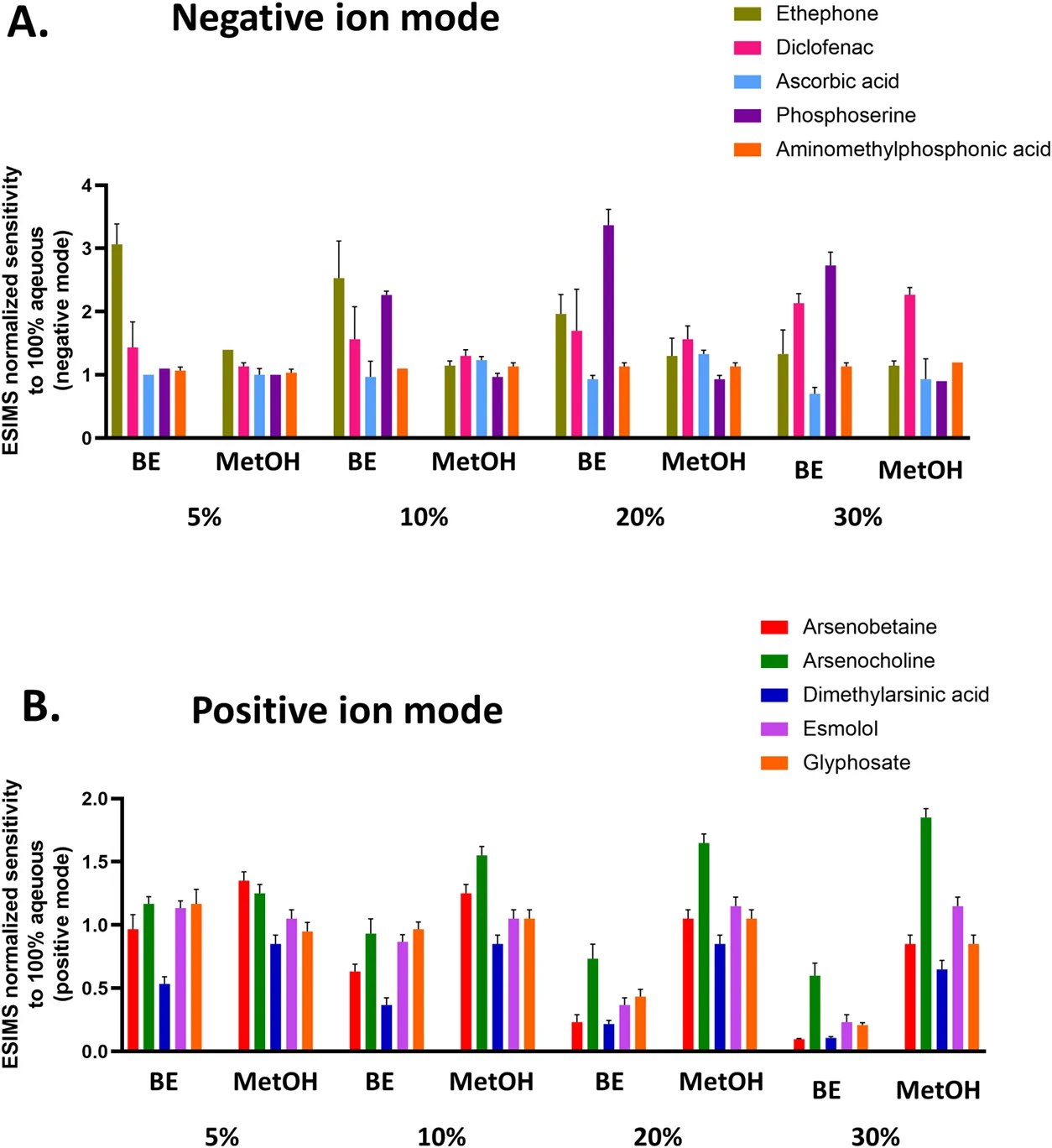
Impact of butoxyethanol on sensitivity of detection by electrospray ionization mass spectrometry (Agilent Ultivo triple quadrupole LC/MS). The graphs below show sensitivity in the negative-ion mode (A) and in the positive-ion mode (B). The experiments were performed using model compounds with different ionization properties (indicated in the figure). To facilitate comparison between butoxyethanol (BE) and methanol (MetOH), data was normalized to 100% aqueous (0.1% v/v formic acid) as a reference point. ESI source parameters: nebulizer gas temperature and flow 350 °C and 10 L min^–1^; sheath gas temperature and flow 400 °C and 12 L min^–1^; nebulizer pressure 35 psi; capillary voltage 3000 V.

Butoxyethanol is a common organic solvent widely used in a variety of applications, such as household cleaners, paints, nail polish, and enamel removers, at concentrations up to 50% v/v. Indeed, butoxyethanol was previously used as a solvent for the extraction of small-molecule arsenic species from peanut butter^22^ and the determination of total palladium in pharmaceutical dosage form.^23^ Butoxyethanol has relatively low toxicity (LD_50_ 2.5 g kg^–1^ compared with 1.2–2.7 g kg^–1^ for methanol), low potential for bioaccumulation, and rapid biodegradability with a half-life of 7–28 days catalyzed by aerobic bacteria to carbon dioxide and water as end products.^24^ Therefore, the eluent has a favorable profile compared with other eluents in reversed-phase liquid chromatography, such as tetrahydrofuran, dichloromethane, and toluene, which have been used as additives where 100% methanol/acetonitrile is not sufficient for fast elution. Furthermore, due to its high elution strength, it can be used in largely aqueous mobile phases unlike acetonitrile and methanol, which minimizes organic solvent consumption.

Butoxyethanol is miscible with water in all proportions below a lower critical solution temperature (LCST) of ca. 49°C.^25^ However, prolonged testing showed no impact when using a 60 °C column temperature at concentrations up to 50% v/v in water, possibly due to short column transition time. As mentioned above, butoxyethanol has significantly lower viscosity than hexanediol. However, a 30% v/v concentration of this eluent yielded ca. 1.5-fold higher backpressure than 50% v/v of methanol under identical conditions, and therefore, we recommend employing column temperatures ≥45°C.

In conclusion, butoxyethanol is a novel, biodegradable, and exceptionally strong eluent in liquid chromatography that provides advantages general to reversed-phase liquid chromatography and specific to ICPMS chromatographic detection. It shows superior performance relative to hexanediol in terms of elution strength and applicability for long UHPLC columns due to its significantly lower viscosity. The use of this eluent is another step forward in eliminating the need for the organic ICPMS mode in order to facilitate coupling this detector with reversed-phase chromatography and unlock new applications at previously unachievable sensitivity levels in speciation analysis.

## Data Availability

All relevant data was included in the manuscript. Additional data can be made available on request. No custom code was used to generate or process the data described in this manuscript.
